# The Aberrant Expression of the Mesenchymal Variant of FGFR2 in the Epithelial Context Inhibits Autophagy

**DOI:** 10.3390/cells8070653

**Published:** 2019-06-29

**Authors:** Monica Nanni, Danilo Ranieri, Flavia Persechino, Maria Rosaria Torrisi, Francesca Belleudi

**Affiliations:** 1 Laboratory affiliated to Istituto Pasteur Italia—Fondazione Cenci Bolognetti, Department of Clinical and Molecular Medicine, Sapienza University of Rome, 00185 Rome, Italy; 2 S. Andrea University Hospital, 00189 Rome, Italy

**Keywords:** FGFR2c, autophagy, keratinocyte, MTOR, JNK1

## Abstract

Signaling of the epithelial splice variant of fibroblast growth factor receptor 2 (FGFR2b) triggers both differentiation and autophagy, while the aberrant expression of the mesenchymal FGFR2c isoform in epithelial cells induces impaired differentiation, epithelial mesenchymal transition (EMT) and tumorigenic features. Here we analyzed in the human keratinocyte cell line, as well as in primary cultured cells, the possible impact of FGFR2c forced expression on the autophagic process. Biochemical and quantitative immunofluorescence analysis, coupled to the use of autophagic flux sensors, specific substrate inhibitors or silencing approaches, showed that ectopic expression and the activation of FGFR2c inhibit the autophagosome formation and that AKT/MTOR is the downstream signaling mainly involved. Interestingly, the selective inhibition of AKT or MTOR substrates caused a reversion of the effects of FGFR2c on autophagy, which could also arise from the imbalance of the interplay between AKT/MTOR pathway and JNK1 signaling in favor of JNK1 activation, BCL-2 phosphorylation and possibly phagophore nucleation. Finally, silencing experiments of depletion of ESRP1, responsible for FGFR2 splicing and consequent FGFR2b expression, indicated that the switching from FGFR2b to FGFR2c isoform could represent the key event underlying the inhibition of the autophagic process in the epithelial context. Our results provide the first evidence of a negative impact of the out-of-context expression of FGFR2c on autophagy, suggesting a possible role of this receptor in the modulation of the recently proposed negative loop between autophagy and EMT during carcinogenesis.

## 1. Introduction

The fibroblast growth factor receptors (FGFR1-4) are four receptor tyrosine kinases regulating key processes, such as cell proliferation, differentiation, migration and survival [1,2]. The alternative splicing of the IgIII loop in FGFR1-3, generates the FGFRIIIb or the FGFRIIIc isoforms, which are mainly expressed in epithelial and mesenchymal tissues, respectively [3]. Deregulation of FGF/FGFR signaling can play either oncogenic or tumor suppressive roles [2,4]. In this regard, the epithelial isoform of FGFR2 (FGFR2b) is a well-recognized regulator of epidermal differentiation and skin homeostasis [5,6,7] exerting a tumor suppressive role in vitro and in vivo [8,9]. According to these studies, our group has demonstrated that FGFR2b controls the entire program of human keratinocyte differentiation [10,11,12] and that PKCδ and PKCα signaling downstream FGFR2b are involved in different steps of this process [12]. However, we also found that, in the same epidermal tissue context, the altered FGFR2 splicing and the aberrant expression of the mesenchymal FGFR2c isoform induces changes in the specificity for FGFs, leading to impairment of differentiation [13], epithelial mesenchymal transition (EMT) and early tumorigenic features [14]. The observation that FGFR2b/FGFR2c switching is also induced in keratinocytes by the E5 oncoprotein of human papillomavirus 16 (HPV16E5) [15], which is expressed in the early stages of virus infection, further supports the hypothesis that FGFR2c aberrant expression might be a precocious event in epithelial tumorigenesis.

At the light of growing evidences showing that FGFs would control cell differentiation by regulating autophagy in several tissues [16,17,18], we have also demonstrated that FGFR2b signaling triggers the autophagic process in human keratinocytes, showing that FGFR2b-induced autophagy and receptor-mediated early differentiation are interplaying events [19,20,21] and that JNK1 is the downstream signaling pathway at the crossroad between them [21,22].

However, autophagy not only regulates several biological functions, such as cell differentiation, but can also play either onco-suppressive or oncogenic roles in cancer, depending on its stage. In particular, during the initial steps of tumorigenesis autophagy, it appears to be linked to a negative loop to EMT, in established tumors this process has a pro-survival effect and its possible interplay with EMT remains still debated [23,24,25]. Therefore, keeping in mind the role of FGFR2c in driving EMT, here we pointed on to establish if and how the aberrant expression of FGFR2c could impact on autophagy. The results obtained showed that FGFR2c expression and signaling in epithelial context negatively interfere with the autophagic process, suggesting that this interference could significantly contribute to cancerogenesis.

## 2. Results

### 2.1. FGFR2c Expression and Signaling Inhibit Autophagy in Human Keratinocytes

**Figure 1:**
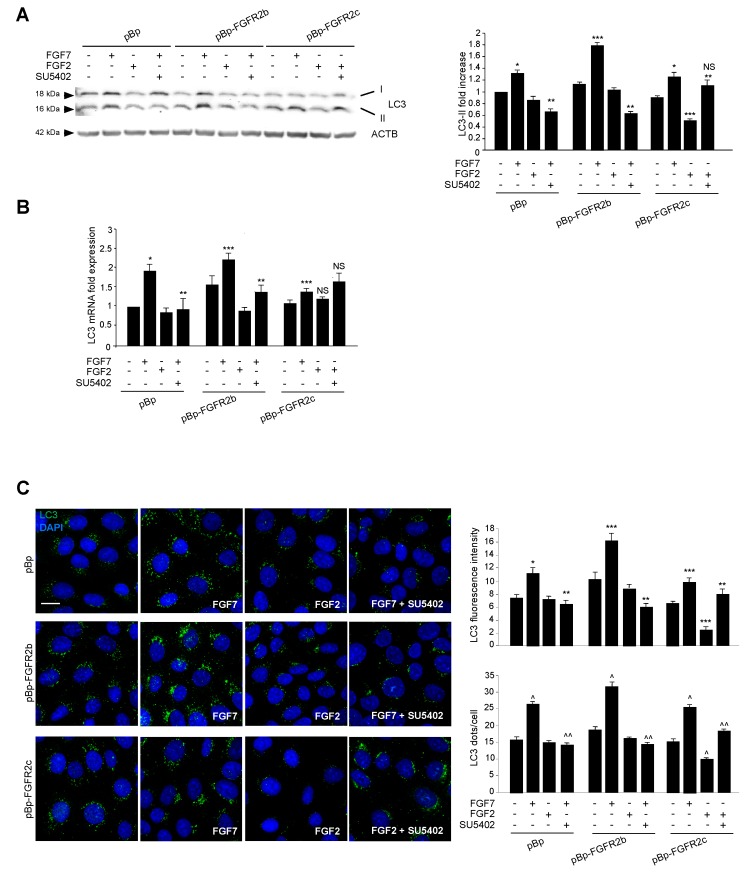
The ectopic expression of FGFR2c and its signaling inhibit autophagy in human keratinocytes. HaCaT cells, stably transduced with pBp-FGFR2b or pBp-FGFR2c constructs or with the empty pBp retroviral vector as control, were left untreated or stimulated with FGF7 or FGF2 in presence or absence of the FGFR2 tyrosine kinase inhibitor SU5402 as described in Material and Methods. (**A**) Western blot analysis showed that, while FGF7 stimulation increases the levels of LC3-II in all clones, FGF2 stimulation significantly decreased them only in HaCaT pBp-FGFR2c cells. All the observed effects were abolished by the presence of SU5402. Equal loading was assessed with the anti-ACTB antibody. For the densitometric analysis the values from three independent experiments were normalized and expressed as fold increases and are reported as mean values ± standard deviations (SD). Student’s *t* test was performed, and significance levels are defined as *P* < 0.05. * *p* < 0.05 and *** *p* < 0.001 vs the corresponding FGF-unstimulated cells; ** *p* < 0.05 vs the corresponding SU5402-untreated cells; not significant (NS) vs the corresponding FGF-unstimulated, SU5402-untreated cells. (**B**) Real-time Reverse Transcriptase-Polymerase Chain Reaction (RT-PCR) analysis shows that while FGF7 stimulation induces the increases of LC3 mRNA transcripts in all clones, FGF2 treatment does not affect them. The results observed in HaCaT pBp and pBp-FGFR2b upon FGF7 stimulation were abolished by SU5402. Results are expressed as mean values ± SE. Student’s *t* test was performed, and significance levels were defined as *P* < 0.05. * *p* < 0.01, *** *p* < 0.05 and NS vs the corresponding FGF-unstimulated cells; ** *p* < 0.05 and NS vs the corresponding SU5402-untreated-cells. (**C**) Quantitative immunofluorescence analysis shows that LC3 signal intensity was increased by FGF7 stimulation in all clones, but it appears strongly reduced upon FGF2 treatment only in HaCaT pBp-FGFR2c cells. The observed effects were abolished by SU5402 treatment. Quantitative analysis of the fluorescence intensity and LC3 positive dots per cell were performed as described in Materials and Methods, and the results are expressed as mean values ± standard errors (SE). The student’s *t* test was performed, and significance levels were defined as *P* < 0.05. * *p* < 0.01, *** *p* < 0.001 and ^ *p* < 0.0001, vs the corresponding FGF-unstimulated cells; ** *p* < 0.001 and ^^ *p* < 0.0001 vs the corresponding SU5402-untreated cells.

To analyze the effect of FGFR2c expression and signaling on autophagy and to compare it to that previously described by us for FGFR2b [19,20,21,22], we took advantage of the human keratinocyte HaCaT clones stably transduced with pBp-FGFR2b or pBp-FGFR2c retroviral constructs or with empty pBp vector, as negative control [14]. Cells were left untreated or stimulated with FGF7, the specific ligand of FGFR2b, or with FGF2, which does not bind to FGFR2b, but is able to activate other FGFRs including FGFR2c. Western blot analysis showed that, while FGF7 stimulation increased the levels of the band corresponding to the lipidated form of the microtubule associated protein 1 light chain 3 (MAP1LC3/LC3-II) in all clones (Figure 1A), as expected [19,20,21,22], FGF2 treatment significantly decreased them only in cells ectopically expressing FGFR2c (Figure 1A). Both the FGFR2b-dependent induction and FGFR2c-mediated inhibition of autophagy were abolished by the specific FGFR2 tyrosine kinase inhibitor SU5402 (Figure 1A), demonstrating that, in both cases, the signaling of FGFR2 isoforms was required. Since it is widely accepted that autophagy is not only a post-translationally regulated, but also a transcriptionally controlled process [26] and we have previously shown that FGFR2b signaling plays a role in this transcriptional control [20,21], we wondered whether FGFR2c might impact on autophagy also by affecting the expression of LC3 gene. To this aim, the mRNA transcript levels of LC3, which we have previously demonstrated to be increased in response to FGF7 in keratinocytes [20,21], were estimated by real-time relative RT-PCR. The results showed that, while FGF7 stimulation increased the expression of this gene in all clones (Figure 1B), FGF2 treatment does not affect them, even in HaCaT-pBp FGFR2c (Figure 1B). The impact of FGFR2c aberrant expression and signaling on autophagy was also investigated by immunofluorescence approach. Quantitative immunofluorescence analysis showed that, while LC3 signal intensity, as well as the number of LC3 positive dots per cell, were increased by FGF7 stimulation in all clones (Figure 1C), they appeared reduced in response to FGF2 only in HaCaT pBp-FGFR2c cells (Figure 1C). Again, all the observed effects were abolished by SU5402 (Figure 1C), confirming the requirement of FGFR2 isoform activation. Thus, the ectopic expression of FGFR2c and its signaling, which is known to exert an oncogenic outcome in human keratinocytes, appear also to negatively impact on autophagy.

### 2.2. The Autophagosome Formation is the Autophagic Step Impaired by FGFR2c Expression and Signaling

**Figure 2:**
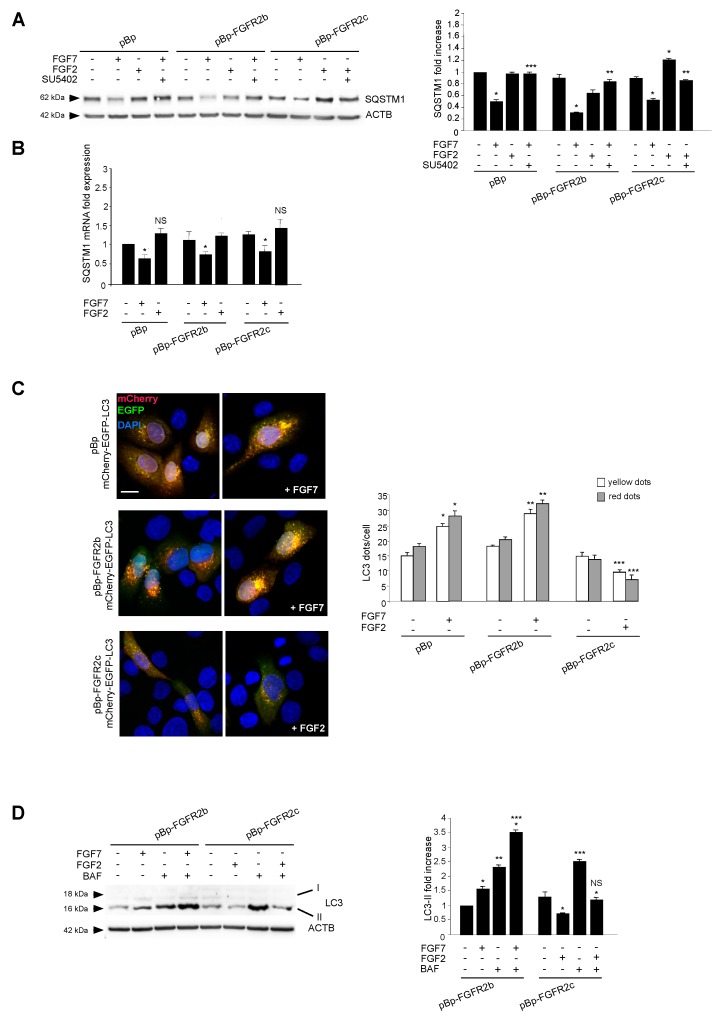
FGFR2c expression and signaling inhibits the autophagosome formation. (**A**) HaCaT pBp, pBp-FGFR2b or pBp-FGFR2c cells were left untreated or stimulated with FGF7 or FGF2 in presence or absence of the FGFR2 tyrosine kinase inhibitor SU5402 as above. Western blot analysis shows that, the 62 kDa band corresponding to SQSTM1 was significantly increased only in HaCaT pBp-FGFR2c clones after FGF2 stimulation, while it decreases in all clones upon FGF7 stimulation. All the observed effects were abolished by SU5402. Equal loading was assessed with anti-ACTB antibody. Densitometric analysis and the student’s *t* test were performed as reported above. * *p* < 0.05 vs the corresponding FGF-unstimulated cells; ** *p* < 0.05 and *** *p* < 0. 001 vs the corresponding SU5402-untreated cells. (**B**) HaCaT clones were stimulated with FGFR2 ligands as above. Real-time RT-PCR analysis shows that FGF7 stimulation decreased SQSTM1 mRNA transcripts in all clones, while FGF2 treatment did not affect them. Results are expressed as mean values ± SE. The student’s *t* test was performed, and significance levels were defined as *P* < 0.05. * *p* < 0.01 vs the corresponding FGF7-unstimulated cells; NS vs the corresponding FGF7-unstimulated cells. (**C**) HaCaT clones were transiently transfected with mCherry-EGFP-LC3 construct. Cells were then left untreated or stimulated with FGF7 or FGF2 as above. Quantitative fluorescence analysis showed that, while FGF7 stimulation increased both yellow and red dots in HaCaT pBp and HaCaT pBp-FGFR2b cells, FGF2 stimulation decreased them in HaCaT pBp-FGFR2c cells. Quantitative analysis was performed as described in Materials and Methods, and results were expressed as mean values ± SE. The student’s *t* test was performed as reported in the legend to Figure 1B. * *p* < 0.05 and ** *p* < 0.001 vs the corresponding FGF7-unstimulated cells; *** *p* < 0.01 vs the corresponding FGF2-unstimulated cells. (**D**) HaCaT pBp-FGFR2b and pBp-FGFR2c clones were left untreated or stimulated with FGF7 or FGF2 in the presence or absence of bafilomycin A1 for the last 3 h. Western blot analysis showed that the increase in the levels of LC3-II upon FGF7 stimulation observed in HaCaT pBp-FGFR2b was further enhanced by bafilomycin while the decrease of LC3-II observed in HaCaT pBp-FGFR2c cells upon FGF2 stimulation was not recovered in the presence of the drug. Equal loading was assessed with anti-ACTB antibody. Densitometric analysis and the student’s *t* test were performed as reported above. * *p* < 0.05 vs the corresponding FGF-unstimulated cells; ** *p* < 0.05, *** *p* < 0.01 and NS vs the corresponding bafilomycin-untreated cells.

The amount of intracellular autophagosomes usually depends on the balance between their formation and their lysosomal-mediated degradation. Therefore, in order to assess how the ectopic FGFR2c could impact on the autophagic flux, the levels of the well-known autophagy substrate SQSTM1/p62 (sequestosome 1) was estimated by Western blot analysis. The evident decrease of the 62 kDa band corresponding to SQSTM1, observed in all clones upon FGF7 stimulation (Figure 2A), confirmed the ability of FGFR2b signaling to trigger mainly the autophagosome assembly. In contrast, the significant increase of the SQSTM1 band, observed exclusively in HaCaT pBp-FGFR2c clones and only in response to FGF2 (Figure 2A), indicated that FGFR2c signaling might act via the inhibition of new autophagosome formation, rather than by accelerating their turnover. The observed effects were abolished by SU5402 (Figure 2A), confirming the requirement of receptor isoform activation. Since it is well known that SQSTM1 can be also transcriptionally regulated under conditions that modulate autophagy, we also investigated its mRNA expression levels in HaCaT clones stimulated as above. The results showed that FGF7 stimulation induced an evident decrease of SQSTM1 mRNA transcripts in all clones (Figure 2B), while FGF2 treatment did not significantly impact on them (Figure 2B). The ability of FGFR2c to negatively interfere with the phagosome formation, rather than their turnover, was also investigated using fluorescence approaches, transfecting HaCaT clones with a pDest-mCherry-EGFP-LC3 tandem construct [27]. In fact, mCherry-EGFP-LC3 is an autophagic flux sensor, since EGFP fluorescence (green) is quenched in acidic environments, whereas mCherry (red) is an acidic-stable fluorescent tag: The nascent autophagosomes are both red and green (yellow) labeled, whereas the acidic autolysosomes appear red, as a consequence of the EGFP quenching. Quantitative fluorescence analysis, performed on transfected cells left untreated or stimulated with FGFR2 ligands as above, showed that, while FGF7 stimulation increased both yellow and red dots (corresponding to autophagosomes and autophagolysosomes, respectively) (Figure 2C), FGF2 treatment significantly decreased them in HaCaT pBp-FGFR2c cells (Figure 2C). These results further confirmed that FGFR2c activation appear to inhibit new autophagosome assembly.

Finally, we monitored the LC3-II levels in the presence or absence of the well-known inhibitor of the autophagosome-lysosome fusion bafilomycin A1. Western blot analysis showed that the effect of increase of LC3-II band generally induced by this drug (Figure 2D) was not found in HaCaT pBp-FGFR2c upon FGF2 stimulation (Figure 2D). These findings demonstrated that the observed decrease of LC3-II induced by FGF2 in HaCaT pBp-FGFR2c cannot be ascribed to an acceleration of the autophagic flux.

Since we have previously shown that FGFR2b signaling plays a role in the transcriptional control of several ATG genes, other than LC3 [20,21], we also assessed whether FGFR2c might impact on autophagy by affecting some of these genes To this aim, the mRNA transcript levels of the BECN1 and ATG5 were estimated by real-time RT-PCR. The results showed that FGF2 treatment did not affect them, even in HaCaT-pBp FGFR2c (Figure S1). Therefore, we feel confident to conclude that the interference exerted by FGFR2c on autophagy does not occur at the transcriptional level.

### 2.3. AKT/MTOR is the Downstream Pathway Responsible for FGFR2c-Mediated Inhibition of Autophagy

**Figure 3:**
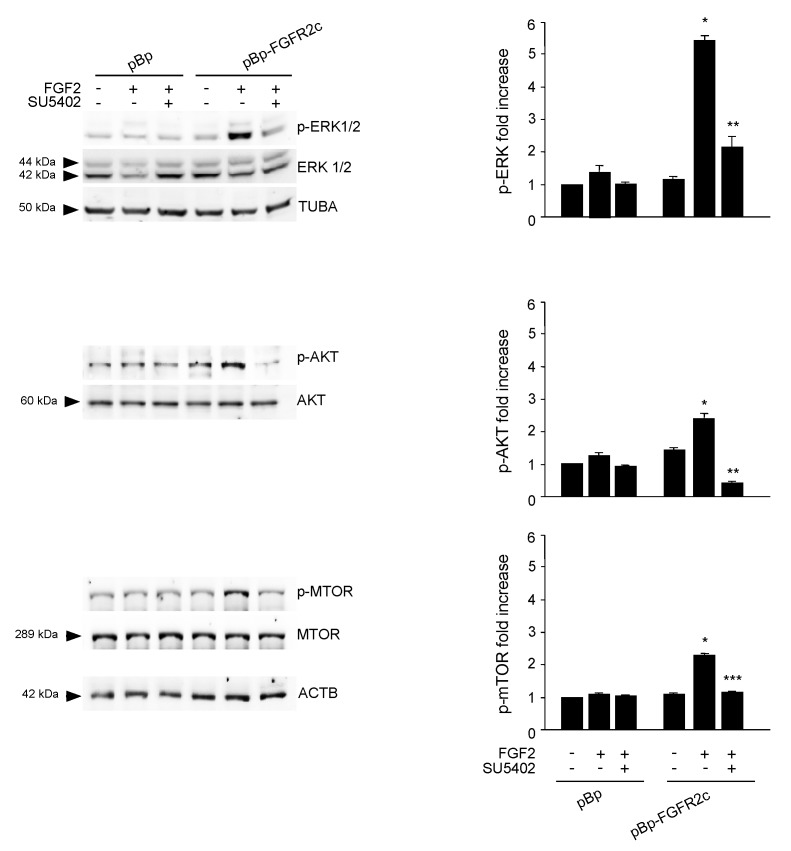
FGF2 stimulation activates ERK1/2 and AKT/MTOR signaling pathways in HaCaT pBp-FGFR2c. HaCaT pBp and HaCaT pBp-FGFR2c clones were left untreated or stimulated with FGF2 in the presence or absence of the FGFR2 tyrosine kinase inhibitor SU5402, as above. Western blot analysis performed using an antibody directed against the phosphorylated form of ERK1/2, AKT and MTOR demonstrates the activation of each substrates only in HaCaT pBp-FGFR2c upon FGF2 stimulation. These effects were abolished by SU5402 treatment. Equal loading was assessed with anti-ERK1/2, anti-AKT, and anti-MTOR antibodies. Densitometric analysis and Student’s *t* test were performed as reported above. * *p* < 0.05 vs the corresponding FGF2-unstimulated cells; ** *p* < 0.05 and *** *p* < 0.01 vs the corresponding SU5402-untreated cells.

**Figure 4:**
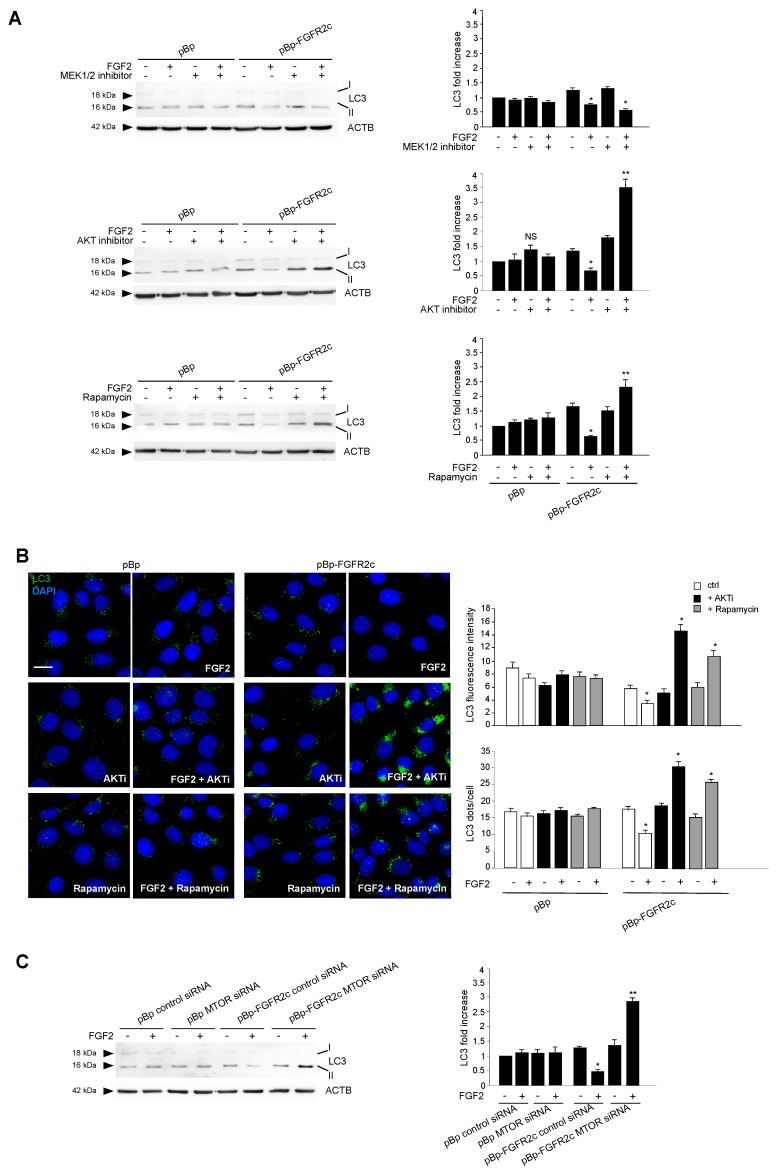
AKT/MTOR is the signaling pathway downstream FGFR2c responsible for FGFR2c-mediated inhibition of autophagy. (**A**) HaCaT pBp and HaCaT pBp-FGFR2c clones were left untreated or stimulated with FGF2 in the presence or absence of the indicated substrate inhibitors as reported in Materials and Methods. Western blot analysis shows that both AKT inhibitor and rapamycin increased the level of LC3-II upon FGF2 stimulation in pBp-FGFR2c cells compared to the corresponding unstimulated cells, while MEK1/2 inhibitor shows no effects. No effects of all the inhibitors are detectable in pBp-FGFR2c cells not stimulated with FGF2 or in pBp control cells. Equal loading was assessed with anti-ACTB antibody. Densitometric analysis and the student’s *t* test were performed as reported above. * *p* < 0.05 vs the corresponding FGF2-unstimulated cells; ** *p* < 0.05 and NS vs the corresponding substrate inhibitor-untreated cells. (**B**) HaCaT pBp and HaCaT pBp-FGFR2c clones were left untreated or stimulated with FGF2 in the presence or absence of AKT inhibitor or rapamycin as reported in Materials and Methods. Quantitative immunofluorescence analysis showed that both AKT inhibitor and rapamicyn reversed the inhibitory effect of FGF2 on LC3 signal intensity and on LC3 positive dots formation only in HaCaT pBp-FGFR2c clones. Quantitative analysis was performed as described in Materials and Methods, and results are expressed as mean values ± SE. The student’s *t* test was performed as reported above. * *p* < 0.001 vs the corresponding FGF2-unstimulated cells. (**C**) HaCaT pBp and HaCaT pBp-FGFR2c clones were transiently transfected with MTOR siRNA or an unrelated siRNA as a control. Cells were then left untreated or stimulated with FGF2 as described in Materials and Methods. Western blot analysis shows that the decrease of LC3-II, observed in HaCaT pBp-FGFR2c control siRNA cells upon FGF2 stimulation, was reversed upon MTOR depletion. Equal loading was assessed with anti-ACTB antibody. Densitometric analysis and the student’s *t* test were performed as reported in the legend to Figure 1A. * *p* < 0.05 and ** *p* < 0.01 vs the corresponding FGF2-unstimulated cells.

To search for the possible downstream signaling pathways responsible for FGFR2c-mediated autophagic repression, MAPK/ERK1/2 and AKT/MTOR signaling were first considered, since they represent the main pathways involved in FGFR-mediated inhibition of autophagy [16,28,29,30]. Western blot analysis demonstrated that, only in cells ectopically expressing FGFR2c, FGF2 stimulation triggered ERK1/2, as well as AKT and MTOR phosphorylation (Figure 3); these effects were abolished by the FGFR2 kinase inhibitor SU5402 (Figure 3), confirming their dependence from FGFR2c activation. Then, in order to assess the possible involvement of these two pathways in FGFR2c-mediated repression of autophagy, we took advantage of specific substrate inhibitors: The ERK1/2 upstream substrates MAP2K/MEK1/2 inhibitor PD0325901 [31], the AKT inhibitor AKT-I-1/2 [32] and the widely used MTOR inhibitor rapamycin. The efficiency of each inhibitor was first assayed by Western blot analysis (Figure S2A). Then, we analyzed their effects on LC3-II expression in HaCaT pBp controls and HaCaT pBp-FGFR2c left untreated or stimulated with FGF2, as above. Surprisingly, Western blot analysis showed that, in the presence of either AKT inhibitor or rapamycin, but not of MEK1/2 inhibitor, the decrease of LC3-II levels induced by FGF2 stimulation in pBp-FGFR2c cells was not only recovered, but significantly increased, compared to the corresponding unstimulated cells (Figure 4A). These results indicated that the selective block of AKT/MTOR pathway was able to revert the effects on autophagy. No effects of all the inhibitors were detectable in cells not stimulated with FGF2 or in pBp control cells (Figure 4A), suggesting that, at least in our keratinocyte model, the ERK1/2 or AKT/MTOR shut-off did not significantly interfere with basal autophagy. The crucial role of AKT/MTOR pathway in FGFR2c-mediated inhibition of autophagy was also confirmed by immunofluorescence approaches, demonstrating that FGF2 stimulation dampened LC3 signal intensity, as well as reduced the LC3 positive dots per cell, only in HaCaT pBp-FGFR2c clones (Figure 4B); however, in the presence of the AKT inhibitor or rapamycin, this effect was completely reversed, resulting in a visible increase of the staining and number of LC3 dots (Figure 4B). Thus, AKT/MTOR is the FGFR2c downstream pathway responsible for autophagy inhibition; nevertheless, if this pathway is selectively switched-off, the negative effect of FGFR2c signaling cascade on autophagic process is not only recovered, but even reversed in autophagy induction. In order to further assess the interesting outcome of AKT/MTOR shut-off on FGFR2c-mediated autophagic effects, we carried out specific protein depletion by siRNA. HaCaT pBp and HaCaT pBp-FGFR2c clones were transfected with MTOR siRNA or with an unrelated siRNA, as negative control, and the efficiency of MTOR depletion was checked by Western blot analysis (Figure S2B). After siRNA transfection, cells were left untreated or stimulated with FGF2 as above. Western blot analysis showed that the decrease of LC3-II, evident in HaCaT pBp-FGFR2c control siRNA cells upon FGF2 stimulation (Figure 4C), turned into a clear increase upon MTOR depletion (Figure 4C).

### 2.4. The Reversion of the Effects of FGFR2c on Autophagy Upon the Selective Block of AKT/MTOR Pathway is Accompanied by JNK1 Activation

**Figure 5:**
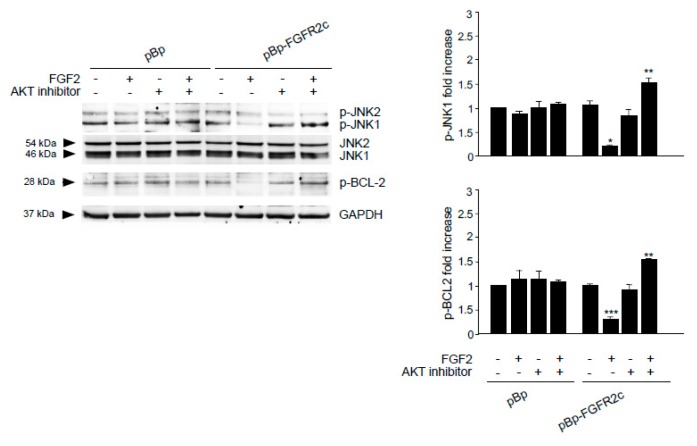
AKT signaling is required for FGFR2c-mediated inhibition of JNK e BCL-2 phosphorylation. HaCaT pBp and HaCaT pBp-FGFR2c clones were left untreated or stimulated with FGF2 in the presence or absence of AKT inhibitor as reported in Materials and Methods. Western blot analysis performed using antibody directed against the phosphorylated form of JNK and BCL-2, demonstrated that FGF2 stimulation decreases the basal phosphorylation of both substrates, while AKT inhibitor reverses this effect. Densitometric analysis and the student’s *t* test were performed as reported in the legend to Figure 1A. * *p* < 0.05 and *** *p* < 0. 01 vs the corresponding FGF2-unstimulated cells; ** *p* < 0.05 vs the corresponding AKT inhibitor-untreated cells.

Searching for the signaling events possibly involved in the reversion of autophagic response to FGFR2c, we focused our attention on MAPK8/JNK1 pathway. In fact, it has been recently proposed that AKT/MTOR signaling exerts an inhibitory function on JNK [33], which is the pathway involved in the induction of autophagy mediated by various FGFRs [17], including FGFR2b [21,22]. In fact, PI3K/AKT/MTOR and JNK signaling are not independent pathways, but a complex network playing important biological roles in cancer [33] and cooperating in the control of different events, including autophagy [34]. Therefore, it is reasonable to suppose that, in our cellular model of keratinocytes ectopically expressing FGFR2c, the induction of autophagy upon AKT/MTOR shut down could be the consequence of the imbalance of the interplay between AKT/MTOR and JNK pathways in favor of JNK1 activation. In order to ascertain it, we checked the phosphorylation levels of JNK1 in the presence of AKT inhibitor: Western blot analysis showed that, in cells expressing FGFR2c, the basal phosphorylation of JNK1 was significantly decreased by FGF2 stimulation (Figure 5), but this effect was reversed by the presence of the AKT inhibitor (Figure 5). Overall, these results suggested that FGFR2c-induced repression of autophagy involves the AKT/MTOR pathway, which also inhibits JNK1 signaling. In fact, upon the selective block of AKT/MTOR pathway, JNK1 phosphorylation/activation increases and contributes to autophagy stimulation.

It is well known that JNK1 signaling triggers the autophagic step of phagophore nucleation via BCL-2 phosphorylation and consequent BECN1 release from the BCL-2/BECN1 inhibitory complex [35,36,37]. This mechanism has been described for the autophagy triggered by FGFR4 [17] and FGFR2b [21]. Therefore, we wondered if FGFR2c activation, which inhibits JNK1 downstream signaling, could also negatively impact on BCL-2 phosphorylation. Western blot analysis showed that the basal phosphorylation of BCL-2 in HaCaT pBp-FGFR2c clones appeared decrease by FGF2 stimulation (Figure 5), but the presence of AKT inhibitor reversed this effect (Figure 5). Thus, while FGFR2c signaling appears to repress autophagy via AKT/MTOR phosphorylation/activation, which also negatively impact on JNK1-mediated phosphorylation of BCL-2, the selective block of AKT/MTOR pathway appears to redirect it toward JNK1 activation, BCL-2 phosphorylation and possibly phagophore nucleation.

### 2.5. Switching From the Epithelial FGFR2b to the Mesenchymal FGFR2c Isoform Underlies the FGF2-Mediated Inhibition of Autophagy in Epithelial Context

**Figure 6:**
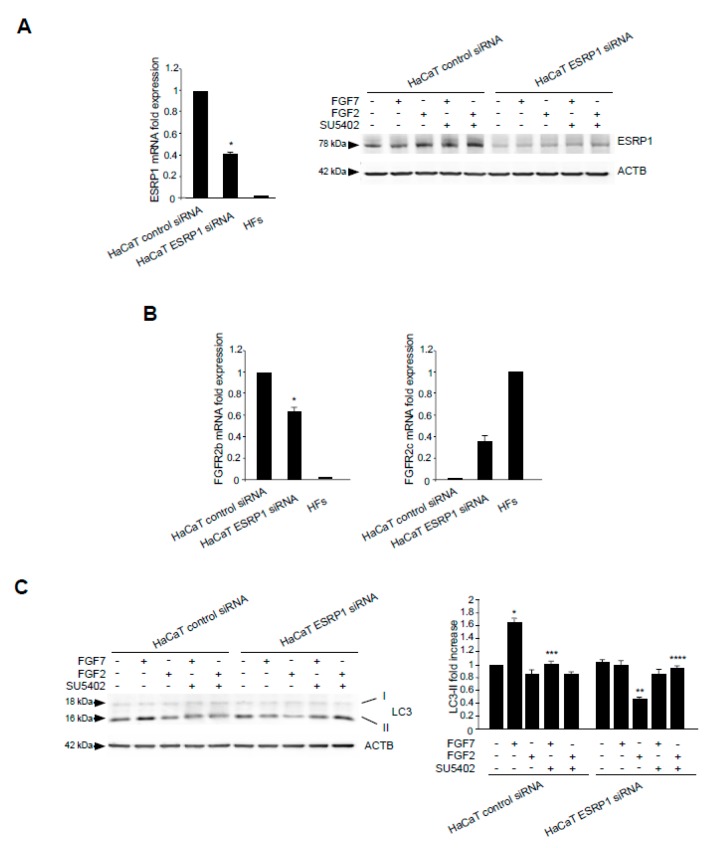

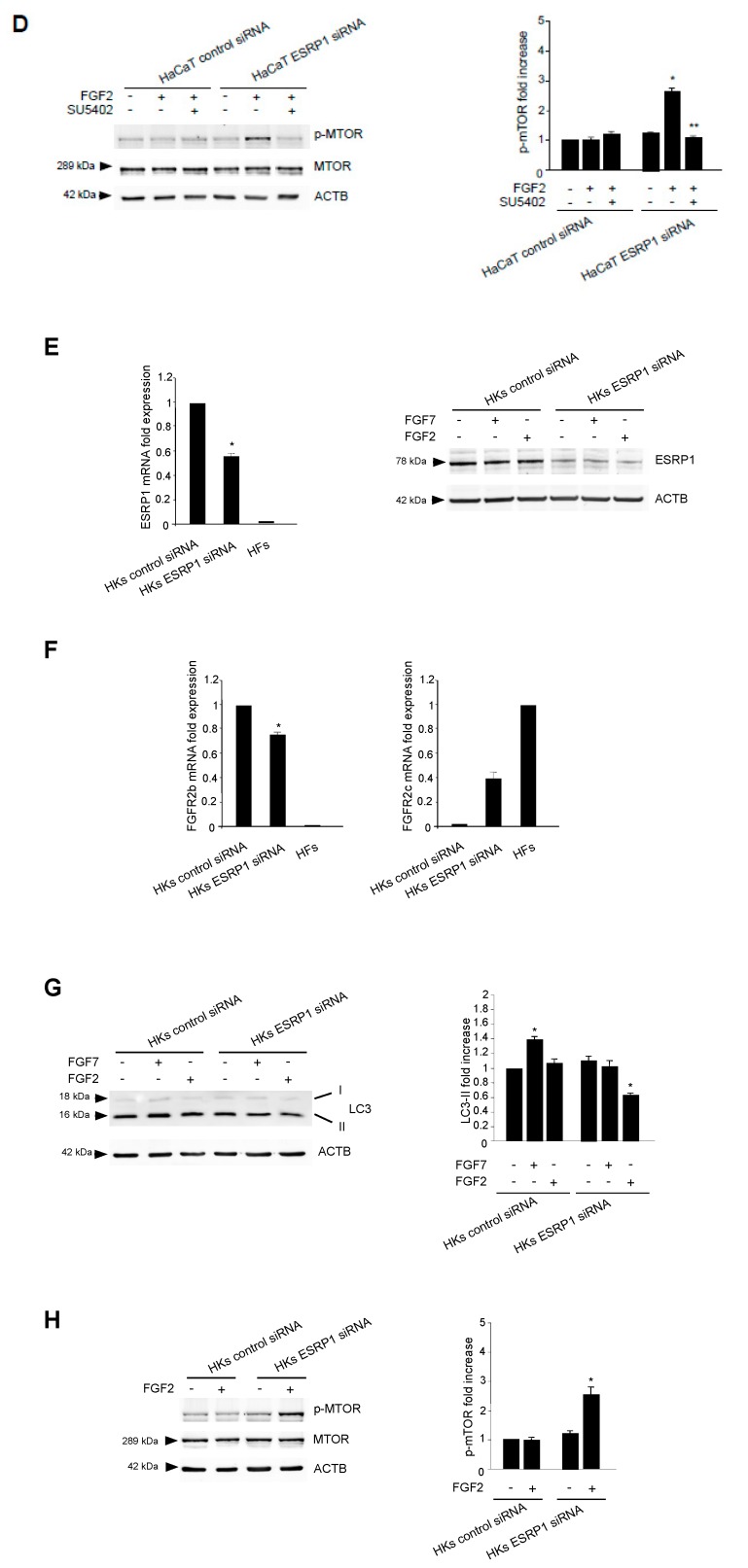
ESRP1 depletion by siRNA in haCaT cells results in aberrant FGFR2 splicing and FGF2-mediated inhibition of autophagy. (**A**) HaCaT cells were transiently transfected with ESRP1 siRNA or an unrelated siRNA as a control. Cells were then left in complete medium (left panel). Alternatively, cells were left untreated or stimulated with FGF7 or FGF2 in the presence or absence of the FGFR2 tyrosine kinase inhibitor SU5402 as above (right panel). Real-time RT-PCR analysis (left panel) and Western blot analysis (right panel) show the efficiency of ESRP1 depletion. Results are expressed as mean values ± SE. The student’s *t* test was performed, and significance levels are defined as *P* values of ± 0.05. * *p* < 0.01 vs the corresponding control siRNA cells. For the Western blot analysis, equal loading was assessed with anti-ACTB antibody. (**B**) HaCaT cells were transiently transfected with ESRP1 siRNA or an unrelated siRNA as a control and then left in complete medium. Real-time RT-PCR analysis shows that ESRP1 depletion leads to a significant decrease of FGFR2b expression (left panel) and to the appearance of FGFR2c (right panel). Results are expressed as mean values ± SE. The student’s *t* test was performed, and significance levels are defined as *P* values of 0.05. * *p* < 0.05 vs the corresponding control siRNA cells. (**C**) HaCaT cells were transiently transfected with ESRP1 siRNA or an unrelated siRNA as a control. Cells were left untreated or stimulated with FGF7 or FGF2 in the presence or absence of the FGFR2 tyrosine kinase inhibitor SU5402 as above. Western blot analysis shows that ESRP1 depletion abolishes the increase of LC3 induced by FGF7 while it induces a decrease of LC3 upon FGF2 stimulation. Equal loading was assessed with anti-ACTB antibody. Densitometric analysis and the student’s *t* test were performed as reported in the legend to Figure 1A. * *p* < 0.05 and ** *p* < 0.01 vs the corresponding FGF-unstimulated cells; *** *p* < 0.01 and **** *p* < 0.05 vs the corresponding SU5402-untreated cells. (**D**) HaCaT cells were transiently transfected with ESRP1 siRNA or an unrelated siRNA as a control and then left untreated or stimulated with FGF2 in presence or absence of SU5402 as above. Western blot analysis performed using antibody directed against the phosphorylated form of MTOR shows the phosphorylation of this substrates upon FGF2 stimulation in HaCaT ESRP1 siRNA cells. Equal loading was assessed with anti-ACTB antibody. Densitometric analysis and the student’s *t* test were performed as reported in the legend to Figure 1A. * *p* < 0.05 vs the corresponding FGF-unstimulated cells; ** *p* < 0. 001 vs the corresponding SU5402-untreated cells. (**E**) HKs cells were transiently transfected with ESRP1 siRNA or an unrelated siRNA as a control and left in complete medium (left panel) or stimulated with FGF7 or FGF2 as above (right panel). Real-time RT-PCR analysis (left panel) and Western blot analysis (right panel) showed the efficiency of ESRP1 depletion. Results are expressed as mean values ± SE. The student’s *t* test was performed, and significance levels are defined as P values of ± 0.05. * *p* < 0.05 vs the corresponding control siRNA cells. For the Western blot analysis, equal loading was assessed with anti-ACTB antibody. (**F**) HKs cells were transiently transfected with ESRP1 siRNA or an unrelated siRNA as a control and then left in complete medium. Real-time RT-PCR analysis showed that ESRP1 depletion lead to a significant decrease of FGFR2b expression (left panel) and to the appearance of FGFR2c (right panel). Results are expressed as mean values ± SE. The student’s *t* test was performed, and significance levels are defined as *P* values of ± 0.05. * *p* < 0.01 vs the corresponding control siRNA cells. (**G**) HKs cells were transiently transfected with ESRP1 siRNA or an unrelated siRNA as a control. Cells were left untreated or stimulated with FGF7 or FGF2 as above. Western blot analysis shows that ESRP1 depletion abolished the increase of LC3 induced by FGF7 while it induced a decrease of LC3 upon FGF2 stimulation. Equal loading was assessed with anti-ACTB antibody. Densitometric analysis and the student’s *t* test were performed as reported in the legend to Figure 1A. * *p* < 0.05 vs the corresponding FGF-unstimulated cells. (**H**) HKs cells were transiently transfected with ESRP1 siRNA or an unrelated siRNA as a control and then left untreated or stimulated with FGF2. Western blot analysis performed using antibody directed against the phosphorylated form of MTOR shows that ESRP1 depletion induces the phosphorylation of this substrates upon FGF2 stimulation. Equal loading was assessed with anti-ACTB antibody. Densitometric analysis and the student’s *t* test were performed as reported in the legend to Figure 1A. * *p* < 0.05 vs the corresponding control siRNA cells.

The epithelial splicing regulatory proteins (ESRPs), and in particular, the ESRP1 isoform are responsible for the FGFR2 splicing and consequent expression of the epithelial FGFR2b isoform [15,38]. Therefore, in order to assess if the FGFR2b versus FGFR2c isoform switching could represent a key event responsible for FGF2-induced inhibition of the autophagic process in epithelial context, we forced this event in keratinocytes, performing ESRP1 depletion by siRNA approach. HaCaT cells where transfected with ESRP1 siRNA (HaCaT ESRP1 siRNA) or with an unrelated siRNA (HaCaT control siRNA), as control, and then stimulated with FGF7 or FGF2 in the presence or not of the FGFR2 kinase inhibitor SU5402, as reported above. The efficiency of ESRP1 depletion was verified through either molecular (Figure 6A, left panel) and biochemical approaches (Figure 6A, right panel), while its effect on FGFR2 isoform expression was quantitated by real-time relative RT–PCR, using human fibroblasts (HFs) as positive control for FGFR2c expression. The results showed that ESRP1 depletion led to a significant decrease of FGFR2b expression (Figure 6B, left panel) and to the appearance of FGFR2c (Figure 6B, right panel), indicating that the correct splicing of the FGFR2 gene, occurring in epithelial context, had been impaired. Then, we focused our attention on the autophagic events. Western blot analysis showed that, the increase of LC3-II in response to FGF7, visible in HaCaT control siRNA cells (Figure 6C) and attributable to FGFR2b activation and signaling, was abolished by ESRP1 depletion (Figure 6C). In contrast, in response to FGF2 stimulation, these HaCaT ESRP1 siRNA cells showed a rate of LC3-II decrease (Figure 6C), as well as of MTOR phosphorylation/activation (Figure 6D) comparable to that observed in HaCaT pBp-FGFR2c cells (see Figure 1A and Figure 3). These results were also confirmed in parallel experiments, using skin-derived primary human keratinocytes (HKs) transfected with ESRP1 siRNA or control siRNA and stimulated with FGF7 or FGF2 as above. Molecular and biochemical approaches were used to verify the efficiency of ESRP1 depletion (Figure 6E), as well as its ability to lead FGFR2b down-regulation and ex-novo expression of FGFR2c (Figure 6F). Finally, Western blot analysis confirmed that ESRP1 depletion was able to abolish the autophagic response of HKs to FGF7 and to sensitize them to FGF2, inducing an LC3-II decrease (Figure 6G) and a MTOR phosphorylation/activation (Figure 6H) comparable to that observed in HaCaT ESRP1 siRNA cells. These results suggested that an altered FGFR2 splicing and the consequent switch from FGFR2b to FGFR2c in epithelial context might drive to autophagy inhibition.

## 3. Discussion

The human genome consists of 20,000–25,000 genes, 95% of which undergo alternative splicing, ensuring the higher proteome diversity [39]. Isoforms arising from the splicing events show distinct and often opposing functions and several studies have suggested that the aberrant splicing represents a crucial event in cancer [39]. The high frequency of splicing aberrations in neoplastic diseases has made necessary new strategies for therapeutic approaches pointed on targeting the aberrant variants and their signaling.

In agreement with the proposed central role of the aberrant splicing in tumorigenesis, the altered splicing of FGFR2 and the consequent appearance of the mesenchymal FGFR2c isoform was observed in several carcinomas [40,41,42,43,44]. Consistent with this clinical evidence, recent studies from our group have demonstrated that the FGFR2 isoform switching and aberrant expression of the mesenchymal FGFR2c isoform in human keratinocytes induces impaired differentiation [13] and EMT [14,15]. The initiation of a pathological type III EMT is also accompanied by the appearance of tumorigenic features [14] indicating that FGFR2 aberrant splicing might represent the precocious event driving the early step of carcinogenesis.

Very recently it is emerging that EMT and autophagy are tumor cell responses to microenvironment stresses occurring in different stages of cancer progression [24,25]. These responses appear to be mutually exclusive and they regulate each other in a complex negative loop [24,25]. However, still many open questions remain to be answered to clarify how tumor cells decide whether to enter one or the other cell stress response. To address tumor cell heterogeneity, the identification of hub molecules able to regulate this intricate interplay appears a promising goal for cancer therapy.Starting from our recent results dealing with the ability of the epithelial FGFR2b isoform in promoting autophagy [19,20,21,22], we speculated that the ectopic FGFR2c might play a central role in the regulation of the negative crosstalk between EMT and the autophagic process in epithelial context. Consistent with this hypothesis, using biochemical, molecular and immunofluorescence approaches, we demonstrated here that the ectopic expression of FGFR2c in normal human keratinocytes efficiently counteracts autophagy. Moreover, forcing the aberrant splicing of FGFR2 in keratinocytes via ESRP1 depletion by siRNA approach, we found that the switching from the epithelial FGFR2b to the mesenchymal FGFR2c isoforms could be the specific event underlying the negative impact on autophagy in epithelial context. This is consistent with the fact that dysregulations in RNA alternative splicing is linked to EMT induction and tumor development [39]. In this regard, RNA splicing regulators, such as ESRPs, are emerging as key proteins playing both oncogenic and tumor suppressive roles via the modulation of RNA isoforms involved in oncogenic signaling pathways [45]. Therefore, we might speculate that, at least in the epidermal context, the down-regulation of ESPRs proteins, responsible for FGFR2 isoform switching, would be the molecular event crucial not only for EMT induction [15] but also for autophagy repression.

**Figure 7:**
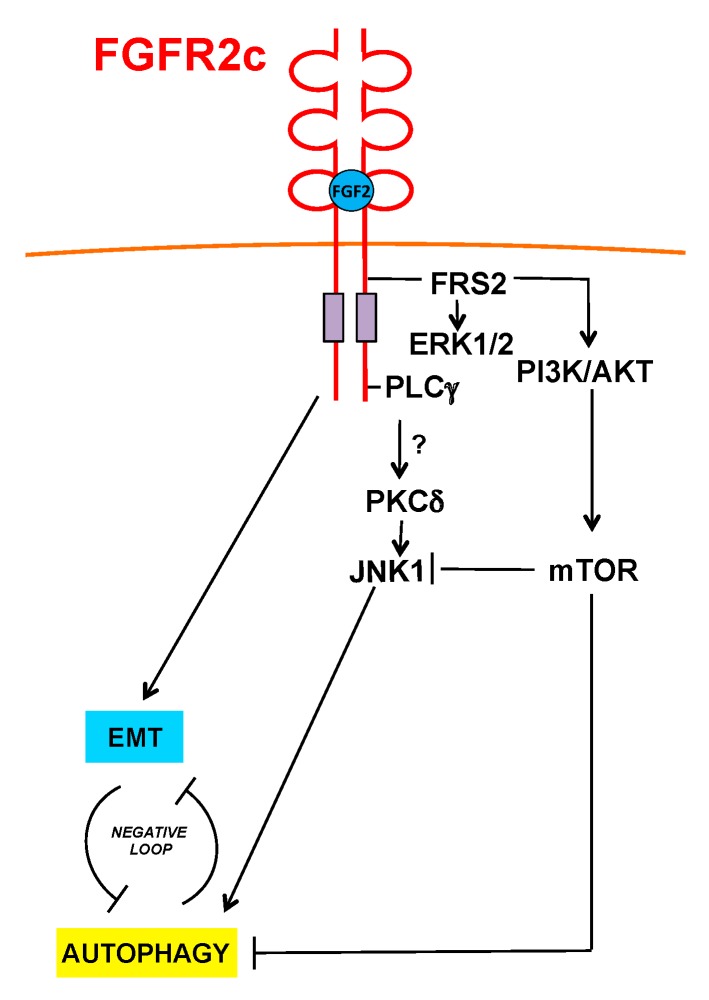
Schematic drawing of the proposed role of FGFR2c and its signaling in the regulation of autophagy and EMT interplay. FGFR2c inhibits autophagy via AKT/MTOR pathway, which also negatively interferes with JNK1 signaling.

Since our results also provide elements to assume that FGFR2c-induced inhibition of the autophagic process is not transcriptionally regulated, we further progressed on the identification of the molecular mechanisms underlying FGFR2c-mediated inhibition of autophagy, identifying AKT/MTOR as the crucial pathway involved (Figure 7). We also observed that the selective inhibition of AKT or MTOR, but not that of MEK1/2, was able not just to dampen, but even to reverse the effects of FGFR2c signaling on autophagy (Figure 7). Interestingly, this unexpected event was accompanied by JNK1 activation. JNK1 signaling is known to be involved in the induction of autophagy mediated by various FGFRs [17], including FGFR2b [21,22] and proceeds via BCL-2 phosphorylation, which in turn allows phagophore nucleation [35,36,37]. Indee, our results showed that both JNK1 and BCL-2 phosphorylation appeared repressed by FGFR2c activation, but strongly activated upon AKT/MTOR shut-off. These findings are in agreement with previous observations of a cooperative crosstalk between PI3K/AKT/MTOR and JNK pathways in the regulation of autophagy also in different cellular contexts, such as PC12 cells and various cancer cell lines [34,46]. In addition, increasing evidences revealed that AKT and JNK pathways interact with each other and that AKT signaling inhibits JNK and different mechanisms have been proposed [33]: In fact, AKT would counteract JNK activation antagonizing the formation of the MAPK8IP1/JIP1-JNK complex [47,48], as well as interfering with the activation of JNK upstream kinases, such as MAP3K5/ASK1, MAP2K4/7/MKK4/7 and MLK [33]. However, since the PI3K/AKT pathway plays essential roles in cancer [33], understanding the molecular mechanisms regulating its crosstalk with JNK may essentially contribute to clarify the specific involvement of each of these pathways in tumor development. Moreover, since autophagy and EMT negatively regulate each other in tumor cells, it is reasonable to suppose that AKT/MTOR inhibition would be able not only to restore autophagy, but also to reverse FGFR2c-induced EMT and tumorigenic features: Further future investigations will be performed in order to address this topic.

Overall, since FGFR2c is simultaneously responsible for the unbalance of the negative crosstalk between autophagy and EMT in favor of this latter, as well as for impairment of differentiation and induction of tumorigenic features, we can conclude that this receptor, expressed as a consequence of an altered splicing event, could be one of the crucial molecular drivers of epithelial deregulation during tumorigenesis. On the other hand, the selective inhibition of specific pathways downstream FGFR2c, such as AKT/MTOR, could represent an effective tool to interfere with the oncogenic autophagy/EMT crosstalk and consequently to counteract the early steps of carcinogenesis.

## 4. Materials and Methods

### 4.1. Cells and Treatments

The human keratinocyte cell line HaCaT, stably expressing FGFR2c (pBp-FGFR2c), overexpressing FGFR2b (pBp-FGFR2b) or the empty vector (pBp) were cultured in Dulbecco’s modified eagle’s medium (DMEM), supplemented with 10% fetal bovine serum (FBS) plus antibiotics. Primary cultures of human keratinocytes and human fibroblasts derived from healthy skin (HKs and HFs, respectively) were obtained from patients attending the Dermatology Unit of the Sant’Andrea Hospital of Rome; all patients were extensively informed and their consent for the investigation was given and collected in written form in accordance with guidelines approved by the management of the Sant’Andrea Hospital. The research was done in agreement with the guidelines of the Helsinki declaration, according to a protocol study approved by the Ethical Committee of Sant’Andrea University Hospital (Prot. CE n. 1591/2013). Primary cells were isolated and cultured as previously described [49,50].

HaCaT clones were transiently transfected with the pDest-mCherry-EGFP tandem expression vector containing LC3 (HaCaT mCherry-EGFP-LC3) [27].

For RNA interference and MTOR or ESRP1 silencing, cells were transfected with MTOR small interfering RNA (MTOR siRNA) (Santa Cruz Biotechnology, Inc., Santa Cruz, CA, USA; SC35409), ESRP1 siRNA (Santa Cruz Biotechnology, SC77526), or an unrelated siRNA as a control, using Lipofectamine 2000 transfection reagent (Life Technologies, Carlsbad, CA, USA; 11668-019) or Fugene HD (Promega, Madison, WI, E2311) according to the manufacturer’s protocol.

For growth factors stimulation, cells were left untreated or incubated with FGF7 (Upstate Biotechnology, Lake Placid, NY, 01-118) or with FGF2 (PeproTech, London, BFGF 100-188) 100 ng/mL for 24 h at 37 °C. To induce activation and signaling of FGFR2 isoforms, cells were serum starved and incubated with FGF7 or FGF2 100 ng/mL for 10 min at 37 °C. For inhibition of FGFR2b and FGFR2c tyrosine kinase activity, cells were pre-incubated with a specific FGFR2 tyrosine kinase inhibitor, SU5402 25 μM (Calbiochem, Nottingham, UK; 572630) for 1 h before treatments with growth factors (GFs).

To inhibit ERK, AKT, or MTOR cells were incubated with the MEK1/2-specific inhibitor PD0325901 (1 μM; Sigma-Aldrich, Saint Louis, MO, USA; PZ0162) AKT-specific inhibitor Akt-I-1/2, (1 μM; Calbiochem, 124005), or with the specific MTOR inhibitor rapamycin (100 nM; Cell Signaling Technology, Beverly, MA, USA; 9904) respectively, for 1 h at 37 °C before being treated with FGF2 in the presence of each inhibitor.

To irreversibly block the fusion between autophagosomes and lysosomes, cells were incubated with bafilomycin A1 (20 nM; Sigma-Aldrich, B1793) for 3 h at 37 °C after treatment with GFs in the presence of the inhibitor.

### 4.2. Immunoflurescence

HaCaT clones, grown on coverslips, were fixed with 4% paraformaldehyde in phosphate-buffered saline (PBS) for 30 min at 25 °C, followed by treatment with 0.1 M glycine for 20 min at 25 °C and with 0.1% Triton X-100 for an additional 5 min at 25 °C to allow permeabilization. Cells were then incubated for 1 h at 25 °C with the following primary antibodies: Mouse monoclonal anti-LC3 (1:100 in PBS, 5F10 Nanotools, Teningen, Germany, 0231). The primary antibodies were visualized using goat anti-mouse IgG-Alexa Fluor 488 (1:200 in PBS, Life Technologies, Carlsbad, CA, A11001) for 30 min at 25 °C. Nuclei were stained with DAPI (1:1000 in PBS; Sigma-Aldrich, D9542). Coverslips were finally mounted with Mowiol (Sigma) for observation. Fluorescence signals were analyzed by scanning cells in a series of sequential sections with an ApoTome System (Zeiss) connected with an Axiovert 200 inverted microscope (Zeiss); image analysis was performed by the Axiovision software (Zeiss) and images were obtained by 3D reconstruction of the total number of the serial optical sections. Quantitative analysis of the fluorescence intensity was performed by the Axiovision software (Zeiss), analyzing 10 different fields randomly taken from 3 independent experiments. Quantitative analysis of LC3-positive dots per cell was performed analyzing 100 cells for each sample in 5 different microscopy fields from 3 different experiments. Results are shown as means ± standard error (SE). The student’s *t* test was performed and significance levels have been defined as *p* < 0.05.

### 4.3. Western Blot Analysis

Cells were lysed in a buffer containing 50 mM HEPES, pH 7.5, 150 mM NaCl, 1% glycerol, 1% Triton X-100, 1.5 mM MgCl2, 5 mM EGTA, supplemented with protease inhibitors (10 g/mL aprotinin, 1 mM phenylmethylsulfonyl fluoride [PMSF], 10 μg/mL leupeptin) and phosphatase inhibitors (1 mM sodium orthovanadate, 20 mM sodium pyrophosphate, 0.5 M NaF). A range of 20 to 50 μg of total protein was resolved under reducing conditions by 8 or 12% SDS-PAGE and transferred to reinforced nitrocellulose (BA-S 83; Schleicher & Schuell, Keene, NH, USA; BA-S83). The membranes were blocked with 5% nonfat dry milk (Bio-Rad Laboratories, Hercules, CA, USA, 170-6404) in PBS 0.1% Tween 20 (Bio-Rad, 170-6531) and incubated with anti-SQSTM1 (BD Bioscience, San Josè, CA, USA, 610833), anti-phospho-JNK (anti-p-JNK) (Thr183/Tyr185, Cell Signaling, 9255S), anti-p-MTOR (Ser 2448, Cell Signaling, 5536S), monoclonal antibodies or with anti-LC3 (MBL, Woburn, MA, PD014), anti ESRP1 (Sigma-Aldrich, HPA023719), anti-p-p44/42 mitogen-activated protein kinase (MAPK) (p-ERK1/2) (Thr202/Tyr204; Cell Signaling, 9101S), anti-p-AKT (Ser 473; Cell Signaling, 9271), anti-p-BCL2 (Ser 70; Cell Signaling, 2827) polyclonal antibodies, followed by enhanced chemiluminescence (ECL) detection (Thermo Scientific, Rockford, IL, USA; 34580).

The membranes were rehydrated by washing in PBS/Tween-20, stripped with 100 mM mercaptoethanol and 2% SDS for 30 min at 55°C and probed again with, anti-AKT (H-136; Santa Cruz Biotechnology, sc-8312), anti-p44/42 MAPK (ERK1/2) (137F5, Cell Signaling, 4695S), anti-JNK (Cell Signaling, 9252S), anti-α-TUBA (Cell Signaling, 2148S) polyclonal antibodies or with anti-MTOR (7C10, Cell Signaling, 2983S), anti-ACTB (Sigma-Aldrich, A5441) monoclonal antibody to estimate the protein equal loading.

Densitometric analysis was performed using Quantity One Program version 4.6.8 (Bio-Rad). The resulting values from three different experiments were normalized, expressed as fold increase respect to the control value and reported in graph as mean values ± standard deviation (SD). The student’s *t* test was performed and significance levels have been defined as *p* < 0.05.

### 4.4. Primers

Oligonucleotide primers necessary for target genes and the housekeeping gene were chosen by using the online tool Primer-BLAST [51] and purchased from Invitrogen (Invitrogen, Carlsbad, CA, USA). The following primers were used: For the MAP1LC3B target gene: 5′-CGCACCTTCGAACAAAGAG-3′ (sense) and 5′-CTCACCCTTGTATCGTTCTATTATCA-3′ (antisense); for the BECN1 target gene: 5′-GGATGGTGTCTCTCGCAGAT-3′ (sense) and 5′-TTGGCACTTTCTGTGGACAT-3′ (antisense); for the ATG5 target gene: 5′-CAACTTGTTTCACGCTATATCAGG-3′ (sense) and 5′-CACTTTGTCAGTTACCAACGTCA-3′ (antisense); for ESRP1 target gene: 5′-GGCTCGGATGAGAAGGAGTT-3′ (sense), 5′-GCACTTCGTGCAACTGTCC-3′ (antisense), for FGFR2b target gene: 5′-CGTGGAAAAGAACGGCAGTAAATA-3′ (sense), 5′-GAACTATTTATCCCCGAGTGCTTG-3′ (antisense); for FGFR2c target gene: 5′- TGAGGACGCTGGGGAATATACG-3 (sense), 5′-TAGTCTGGGGAAGCTGTAATCTCCT 3′ (antisense); for the SQSTM1 target gene: 5′-AGCTGCCTTGTACCCACATC-3 (sense), 5′- CAGAGAAGCCCATGGACAG-3′ (antisense); for the 18S rRNA housekeeping gene: 5′-CGAGCCGCCTGGATACC-3′ (sense) and 5′-CATGGCCTCAGTTCCGAAAA-3′ (antisense). For each primer pair, we performed no-template control and no-reverse-transcriptase control (reverse transcription [RT]-negative) assays, which produced negligible signals

### 4.5. RNA Extraction and cDNA Synthesis

RNA was extracted using the TRIzol method (Invitrogen, Carlsbad, CA, USA; 15596018) according to the manufacturer’s instructions and eluted with 0.1% diethylpyrocarbonate (DEPC)-treated water. Each sample was treated with DNase I (Invitrogen, 18068-015). The total RNA concentration was quantitated by spectrophotometry; 1 μg of total RNA was used for reverse transcription using the iScriptTM cDNA synthesis kit (Bio-Rad, 170-8891) according to the manufacturer’s instructions.

### 4.6. PCR Amplification and Real-Time Quantitation

Real-time RT-PCR was performed using the iCycler real-time detection system (iQ5 Bio-Rad) with optimized PCR conditions. The reactions were carried out in a 96-well plate using iQ SYBR green supermix (Bio-Rad, 1708882), adding forward and reverse primers for each gene and 1 μl of diluted template cDNA to a final reaction mixture volume of 15 μl. All assays included a negative control and were replicated three times. The thermal cycling program was performed as described previously [52]. Real-time quantitation was performed with the help of the iCycler IQ optical system software, version 3.0a (Bio-Rad), according to the manufacturer’s manual. Results are reported as mean values ± SE from three different experiments in triplicate. The student’s *t* test was performed, with significance levels defined as *P* values < 0.05.

## Supplementary Materials

The following are available online at https://www.mdpi.com/2073-4409/8/7/653/s1, Figure S1: FGFR2c-induced inhibition of autophagy is not transcriptionally regulated, Figure S2: Biochemical evaluation of the efficiency of specific signaling pathway substrate inhibitors and siRNAs.

## Abbreviations

ATG5	autophagy-related 5
BCL-2	BCL2 apoptosis regulator
BECN1	Beclin 1, autophagy-related
DAPI	4′,6-diamidino-2-phenylindole
EGFP	enhanced green fluorescent protein
EMT	epithelial-mesenchymal transition
ESRP	Epithelial Splicing Regulatory Protein
FGF2	fibroblast growth factor 2 (basic)
FGF7/KGF	fibroblast growth factor 7
FGFR	fibroblast growth factor receptor
GAPDH	glyceraldehyde-3-phosphate dehydrogenase
HaCaT	human adult skin keratinocytes propagated under low calcium
HFs	Human fibroblasts
HKs	human keratinocytes
HPV16	human papillomavirus type 16
MAP1LC3/LC3	microtubule-associated protein 1 light chain 3
MAP2K/MEK	mitogen-activated protein kinase kinase
MAP2K4/MKK4	mitogen-activated protein kinase kinase 4
MAP2K7/MKK7	mitogen-activated protein kinase kinase 7
MAPK/ERK	mitogen-activated protein kinase
MAPK8/JNK	mitogen-activated protein kinase 8
ACTB	actin beta
MAP3K5/ASK1	mitogen-activated protein kinase kinase kinase 5
MAPK8IP1/JIP1	mitogen-activated protein kinase 8 interacting protein 1
MLK	mixed-lineage kinase
MTOR	mechanistic target of rapamycin
NS	not significant
PI3K	class I phosphoinositide 3 kinase
PKC	protein kinase C
SD	standard deviation
SE	standard error
SDS-PAGE	sodium dodecyl sulphate-polyacrylamide gel electrophoresis
RT-PCR	Reverse Transcriptase-Polymerase Chain Reaction
siRNA	small interfering RNA
SQSTM1/P62	sequestosome 1
TUBA	α-tubulin
