# Supplementary material for: The Aberrant Expression of the Mesenchymal Variant of FGFR2 in the Epithelial Context Inhibits Autophagy
Source: Cells. 2019 Jun 29;8(7):653. doi: 10.3390/cells8070653 (PMC6678203; doi:10.3390/cells8070653)
Supplement: Supplementary file 1 [file cells-08-00653-s001.pdf]

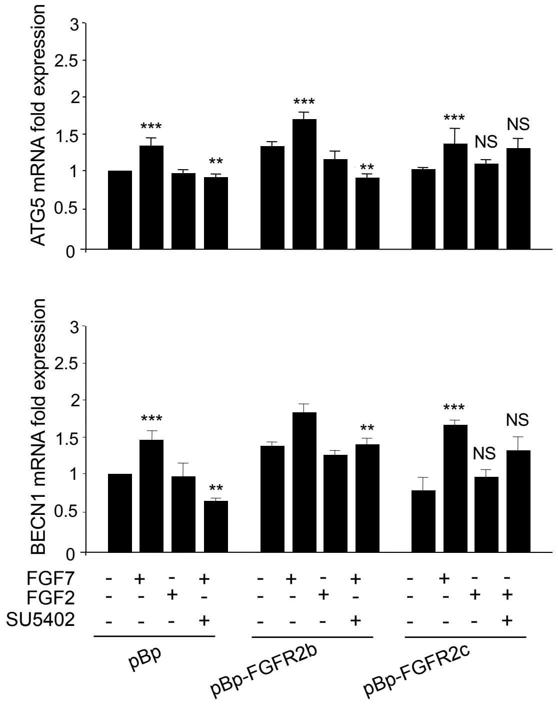

Figure S1

FGFR2c-induced inhibition of autophagy is not transcriptionally regulated.

HaCaT pBp, pBp-FGFR2b or pBp-FGFR2c clones were left untreated or stimulated with FGF7 or FGF2 in presence or absence of the FGFR2 tyrosine kinase inhibitor SU5402 as above. Real-time RT-PCR analysis shows that while FGF7 stimulation induces the increases of ATG5 and BECN1 mRNA transcripts in all clones, FGF2 treatment does not affect them. The results observed in HaCaT pBp and pBp-FGFR2b upon FGF7 stimulation are abolished by SU5402. Results are expressed as mean values ± SE. Student's t test was performed, and significance levels are defined as P < 0.05. *p < 0.01, ***p < 0.05, NS vs the corresponding FGF-unstimulated cells; ** p < 0.05 and NS vs the corresponding SU5402-untreated-cells.

A

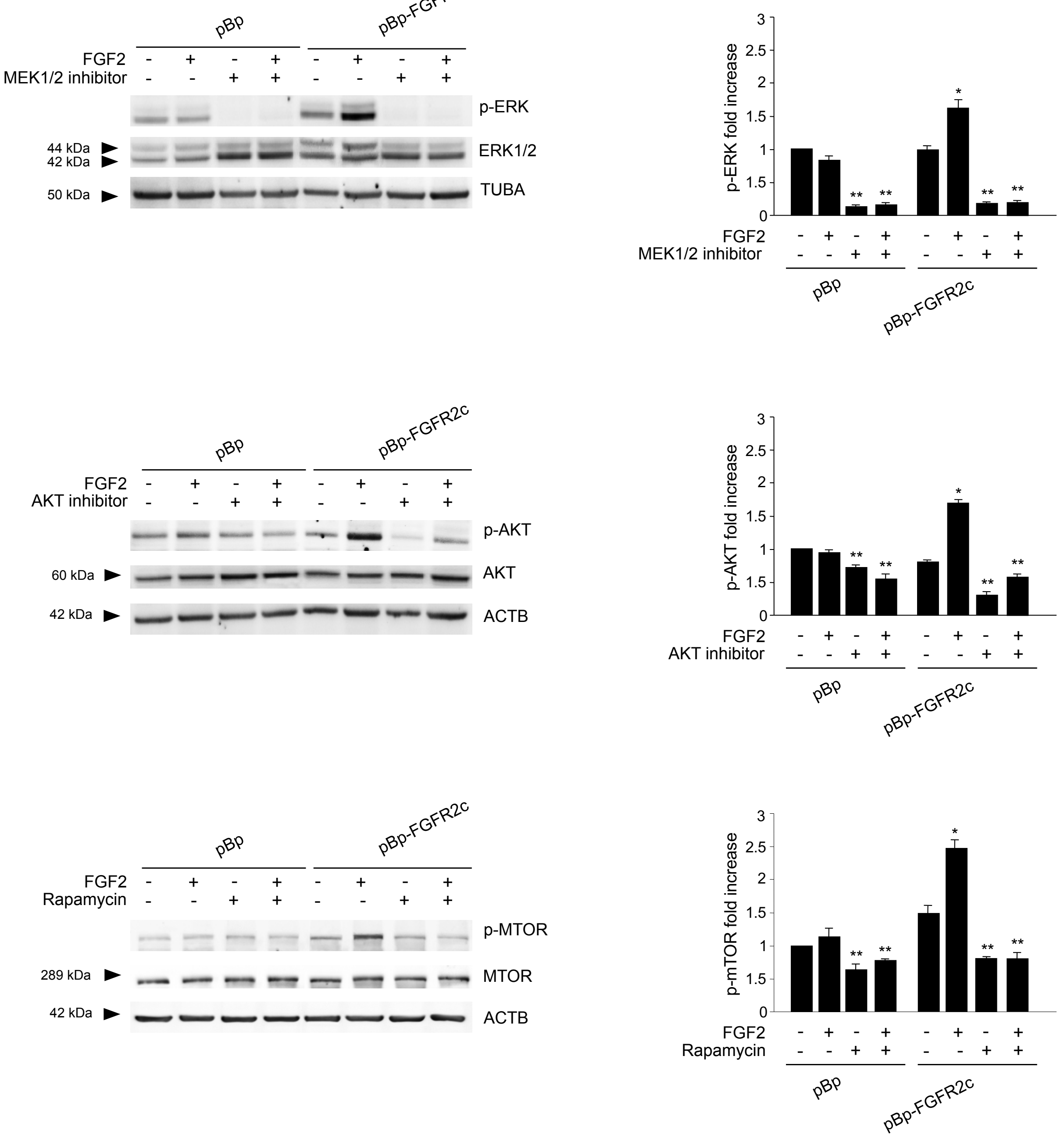
pBp
pBp-FGFR2c
FGF2
MEK1/2 inhibitor
p-ERK
ERK1/2
TUBA
44 kDa
42 kDa
50 kDa
p-ERK fold increase
AKT inhibitor
p-AKT
AKT
ACTB
60 kDa
42 kDa
p-AKT fold increase
Rapamycin
p-MTOR
MTOR
289 kDa
p-mTOR fold increase

B

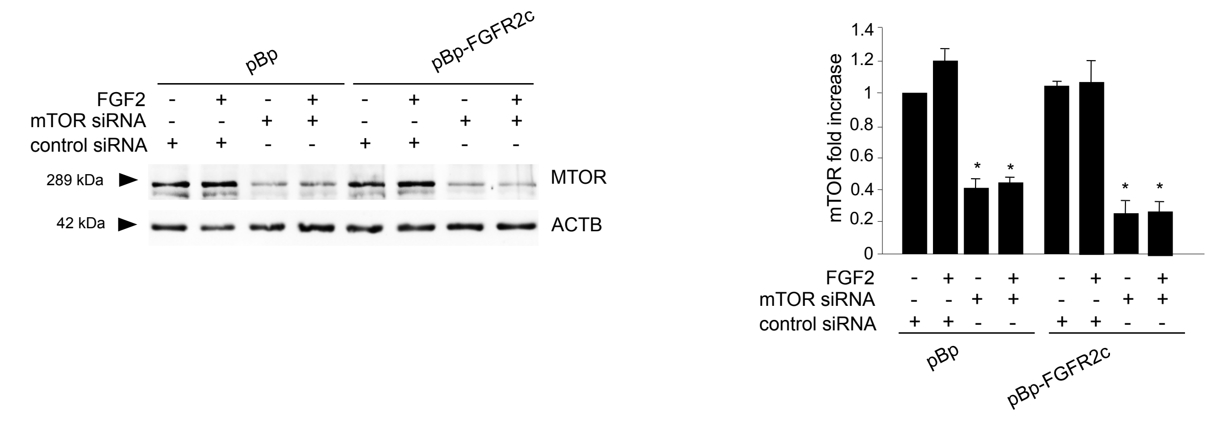
pBp
pBp-FGFR2c
FGF2
mTOR siRNA
control siRNA
MTOR
ACTB
289 kDa
42 kDa
mTOR fold increase

Figure S2

Biochemical evaluation of the efficiency of specific signaling pathway substrate inhibitors and siRNAs

A) HaCaT pBp and HaCaT pBp-FGFR2c clones were left untreated or stimulated with FGF2 in the presence or absence of the indicated substrate inhibitors as reported in Materials and Methods. Western blot analysis performed using antibody directed against the phosphorylated form of each substrate confirms the efficiency of all the inhibitors. Equal loading was assessed with anti-ERK1/2, anti-AKT, and anti-MTOR. For the densitometric analysis, the values from 3 independent experiments were normalized and expressed as fold increases and are reported as mean values ± standard deviations (SD). Student's t test was performed, and significance levels are defined as P values of < 0.05. *p < 0. 05 vs the corresponding FGF2-unstimulated cells; **p < 0.05 vs the corresponding substrate inhibitor-untreated cells B) HaCaT pBp and HaCaT pBp-FGFR2c clones were transiently transfected with MTOR siRNA or an unrelated siRNA as a control. Cells were then left untreated or stimulated with FGF2 as described in Materials and Methods. Western blot analysis shows that MTOR siRNA induces an efficient depletion of MTOR. Equal loading was assessed with anti-ACTB antibody. Densitometric analysis and Student's t test were performed as reported above. *p < 0. 05 vs the corresponding FGF-unstimulated cells.
